# Three-Dimensional Hinge Axis Orientation Contributes to Simultaneous Alignment Correction in All Three Anatomical Planes in Opening-Wedge High Tibial Osteotomy

**DOI:** 10.1016/j.asmr.2024.100888

**Published:** 2024-02-07

**Authors:** Quinten W.T. Veerman, Romy M. ten Heggeler, Gabriëlle J.M. Tuijthof, Feike de Graaff, René Fluit, Roy A.G. Hoogeslag

**Affiliations:** a OCON Centre for Orthopaedic Surgery and Sports Medicine, Hengelo, the Netherlands; b Faculty of Engineering Technology, University of Twente, Enschede, the Netherlands; c Faculty of Science and Engineering, University of Groningen, Groningen, the Netherlands

## Abstract

**Purpose:**

To investigate the simultaneous effect of 3-dimensional (3D) hinge axis (HA) orientation on alignment parameters in all 3 anatomical planes in high tibial osteotomy.

**Methods:**

A computed tomography–based 3D model of a human tibia/fibula was used to establish a 3D tibial coordinate system based on the tibial mechanical axis. In here, an HA was positioned and an opening-wedge high tibial osteotomy with a rotation angle of 10° over the HA was simulated. HA rotation in the axial plane ranged from 0° to 90° and HA tilt relative to the axial plane ranged from –20° to +20°. The study quantified the simultaneous effect of HA orientation on change of alignment parameters in all anatomical reference planes.

**Results:**

HA rotation within the tibial axial plane between orientations perpendicular to the coronal and sagittal planes primarily affected both coronal and sagittal plane alignment, with an inverse relationship between these planes (range: 0°-9.7°); the effect of HA rotation on the change in axial plane alignment was maximally 0.9°. In contrast, HA tilt relative to the tibial axial plane primarily affected axial alignment (maximum change: 6.9°); the effect on change in both coronal and sagittal plane alignment was maximally 0.6°.

**Conclusions:**

HA rotation in the tibial axial plane primarily affects sagittal and coronal plane alignment, and HA tilt relative to the tibial axial plane primarily affects axial plane alignment.

**Clinical Relevance:**

Integrating 3D HA orientation in malalignment planning and correction offers the potential to minimize unintended corrections in nontargeted planes in uniplanar correction osteotomies and to facilitate intentional multiplanar correction with a single osteotomy.

Osteotomy around the knee is an accepted surgical method to correct coronal, sagittal, and axial plane joint malalignment of the leg in patients presenting with unicompartmental osteoarthritis^1^, ^2^, ^3^ or ligamentous and patellofemoral instability^4^, ^5^, ^6^ and to prevent failure of ligamentous reconstruction.^7^^,^^8^

With the current gold standard for preoperative leg malalignment analysis and correction planning, 3-dimensional (3D) space is first simplified into 2-dimensional (2D) radiographs and 2D computed tomography (CT) slices to define 3 (not necessarily orthogonal) 2D planes: a coronal, sagittal, and axial plane.^9^, ^10^, ^11^ Subsequently, malalignment analysis and correction planning are performed per 2-dimensional (2D) plane using a hinge point placed on the radiograph over which the osteotomy is rotated. In 3D, this hinge point is actually a hinge axis (HA) positioned perpendicular to the radiologically depicted 2D anatomical reference plane (Appendix Fig 1).

**Fig 1:**
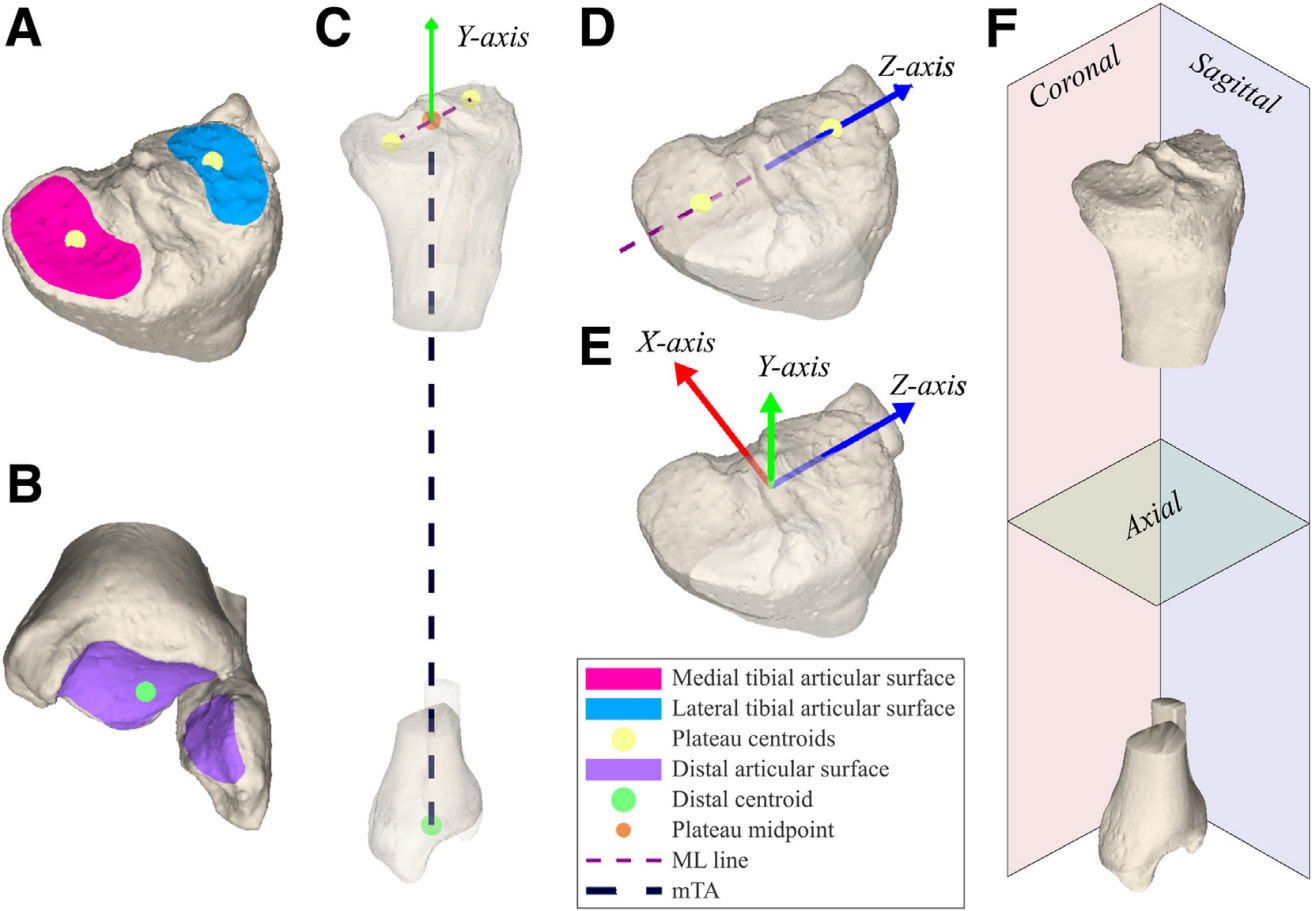
Construction of a clinically relevant mechanical tibial axis-based lower leg coordinate system. (A) The medial and lateral proximal articular surfaces of the tibial plateau were marked, and their centroids were calculated. (B) The distal articular surfaces of the tibia (plafond and medial and lateral malleoli) were marked, and the centroid was calculated. (C) The y-axis (proximal–distal) was defined by the mechanical tibial axis, which was constructed by connecting the point midway between the centroids of the medial and lateral tibial plateaus with the distal centroid. The point midway between the centroids of the medial and lateral tibial plateaus (i.e., the proximal tibial/fibular joint center) was defined as the coordinate system’s origin. (D) The z-axis (medial–lateral) was defined to be perpendicular to the y-axis, in the direction of the line connecting the centroids of the medial and lateral tibial plateaus, and coincident with the coordinate system’s origin. (E) The x-axis (anterior–posterior) was defined by the cross-product of the y- and z-axes. (F) The coronal, sagittal, and axial planes were defined by the y- and z-axes, the y- and x-axes, and the z- and x-axes, respectively. (ML, medial-lateral; mTA, mechanical tibial axis.)

However, the accuracy of osteotomy procedures based on the 2D framework is poor, and osteotomy procedures based on 2D imaging introduce unintended corrections in anatomical reference planes other than the one targeted.^12^, ^13^, ^14^, ^15^, ^16^, ^17^ The reasons for this latter-side effect already have been addressed in previous studies.^18^, ^19^, ^20^, ^21^, ^22^, ^23^ It was demonstrated that when performing an osteotomy, the HA direction must be truly perpendicular to one of the anatomical reference planes in order to leave the alignment parameters in the other 2 anatomical reference planes unaltered. Any rotation of the HA so that it is not perpendicular to 1 of the 3 orthogonal anatomical reference planes would lead to changes in alignment parameter values in more than one anatomical reference plane.^19^^,^^23^ Nevertheless, when using the current osteotomy framework based on 2D imaging, the HA orientation in 3D space is not explicitly enforced.^18^, ^19^, ^20^^,^^22^^,^^23^

By contrast, with the advances in 3D imaging and surgical technologies for malalignment analyses and correction planning and execution,^24^, ^25^, ^26^, ^27^, ^28^, ^29^, ^30^ explicit enforcement of HA orientation in 3D space is possible. Consequently, the effect of 3D HA orientation on change of alignment during osteotomy procedures can not only be used to minimize unintended corrections in nontargeted anatomical reference planes^23^^,^^31^ but also to facilitate intentional correction of alignment in all anatomical reference planes simultaneously.^25^^,^^32^

The purpose of this study is to investigate the simultaneous effect of 3D HA orientation on alignment parameters in all 3 anatomical reference planes in high tibial osteotomy. We hypothesized that (1) rotation of the HA in the axial plane would influence the effect of the osteotomy on alignment parameters in coronal and sagittal plane alignment, with an inverse relationship between these 2 planes; (2) tilting the HA relative to the axial plane would influence the effect of the osteotomy on alignment parameters in the axial plane; and (3) change of location of the HA origin relative to the tibial cortex would influence alignment.

## Methods

An opening-wedge high tibial osteotomy was simulated virtually in a CT-based segmented 3D model of a human tibia/fibula at OCON Centre for Orthopaedic Surgery and Sports Medicine.

### Generation of a 3D Model of the Lower Leg

CT scan data of a healthy contralateral tibia/fibula from 1 patient (44 years; male) who underwent alignment analysis after failure of a primary anterior cruciate ligament reconstruction were retrospectively acquired for use in the study and anonymized. CT acquisition was performed with a Siemens SOMATOM Definition AS (Siemens Healthineers, Erlangen, Germany) with slice thickness = 0.75 mm, kV = 100, eff. mAs = 132 for the knee and 145 for the ankle; kernel = B60s; and field of view: proximal and distal parts of the tibia and fibula around the knee and ankle, respectively. The tibia and fibula were segmented to obtain 3D bone models using Mimics v24.0 (Materialise, Leuven, Belgium).

### Definition of the Lower-Leg Coordinate System and Lower-Leg Alignment Parameters

**Fig 2:**
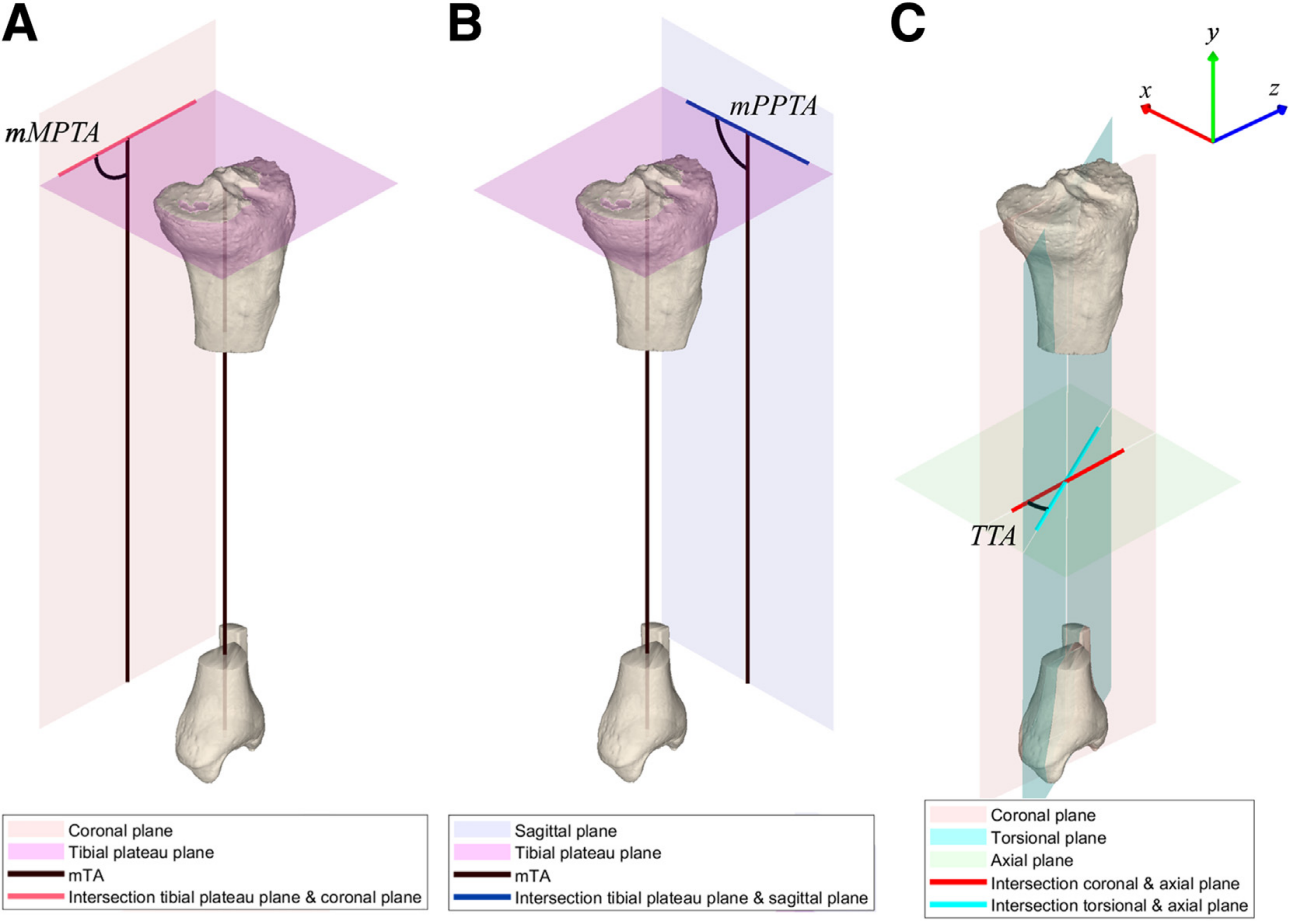
Calculation of 3D lower leg alignment parameters in each of the orthogonal anatomical reference planes. (A) For the coronal plane, varus/valgus alignment was described using the mechanical medial proximal tibial angle (mMPTA), the medial angle between (1) the projection of the mTA on the coronal plane and (2) the intersecting line of the tibial plateau plane with the tibial coronal plane. (B) For the sagittal plane, the posterior tibial slope was described using the mechanical posterior proximal tibial angle (mPPTA) of the tibial plateaus, the posterior angle between (1) the projection of the mTA on the sagittal plane and (2) the intersecting line of the tibial plateau plane with the sagittal plane. (C) For the axial plane, tibial torsion was described using the tibial torsional angle (TTA), the angle between (1) the intersecting line of the coronal plane with the axial plane and (2) the intersecting line of the tibial torsional plane with the axial plane. (3D, 3-dimensional; mTA, mechanical tibial axis.)

A clinically relevant mechanical–tibial axis-based lower leg coordinate system was acquired using 3-matic 16.0 (Materialise; Fig 1).^33^^,^^34^ An average plane of 2 planes fit through the marked medial and lateral tibial plateaus’ articular surfaces (tibial plateau plane; Fig 1A) was obtained to represent the 3D joint orientation of the tibial plateau. Next, the torsional plane was defined by a plane through the proximal tibia/fibula joint center and the most distal points (relative to the coordinate system) of the medial and lateral malleoli.^35^ The effect of change of HA orientation in 3D space on lower leg alignment was illustrated in all 3 anatomical reference planes of the lower leg coordinate system using the following alignment parameters: the proximal tibial joint orientation angle in the coronal plane (varus/valgus alignment) is defined by the mechanical medial proximal tibial angle (mMPTA) between the intersecting line of the tibial plateau’s joint orientation plane with the coronal plane and the projection of the mechanical tibial axis (mTA) on the coronal plane (Fig 2A); the proximal tibial joint orientation angle in the sagittal plane (posterior tibial slope) is defined by the mechanical proximal posterior tibial angle (mPPTA) between the intersecting line of the tibial plateau plane with the sagittal plane and the projection of the mTA on the sagittal plane (Fig 2B); and tibial torsion is defined by the tibial torsional angle between the intersecting lines of the coronal plane and the torsional plane with the axial plane (Fig 2C).

### Simulation of a 3D High Tibial Opening-Wedge Osteotomy

**Appendix Fig 2:**
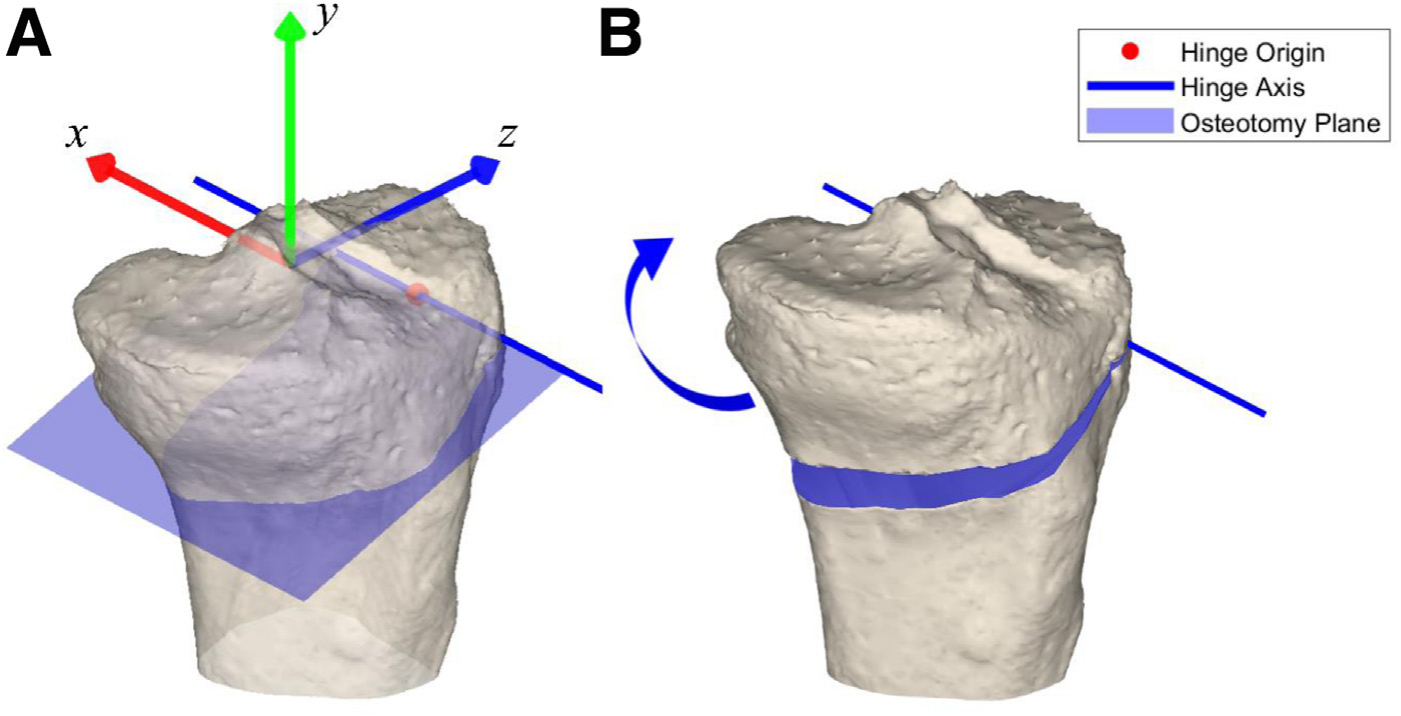
Opening-wedge high tibial osteotomy simulation on a computed tomography–based 3-dimensional model of the tibia. Opening-wedge high tibial osteotomy in the preoperative (A) and postoperative (B) situation after rotation of the proximal tibia 10° over the hinge axis. Hinge axis orientation is at the height of the tip of the fibular head along the longitudinal tibial axis, 10 mm from the lateral tibial cortex, and perpendicular to the tibial coronal plane. The medial entry point of the osteotomy plane was chosen in a surgically feasible location.

An opening-wedge high tibial osteotomy was simulated by placing a HA in the tibial coordinate system and opening the osteotomy over this HA with a predefined rotation angle of 10° (Appendix Fig 2). The orientation of the HA relative to the tibial coordinate system was defined by the HA direction, the position of the HA origin along the longitudinal axis, and the distance of the HA origin to the tibial cortex. Subsequently, the simultaneous effects of stepwise changes in the parameters that define HA orientation on the alignment parameters in all 3 anatomical reference planes were investigated.

An osteotomy can relocate certain landmarks that are used to define the coordinate system and alignment parameters while other landmarks remain stationary. This was taken into account for the simulations.

The simulations consisted of (1) execution of a stepwise change in HA rotation in the axial plane (HA rotation) and HA tilt relative to the axial plane (HA tilt), and (2) execution of a stepwise change in the position of the HA origin along the longitudinal axis and of the distance from the HA origin to the tibial cortex. The simulations, including an update of the coordinate system by recalibration with the relocated landmarks, were performed with in-house code developed using MATLAB r2021b (The MathWorks Inc., Natick, MA).

### Stepwise Change in HA Direction With Rotation and Tilt Relative to the Axial Plane

**Fig 3:**
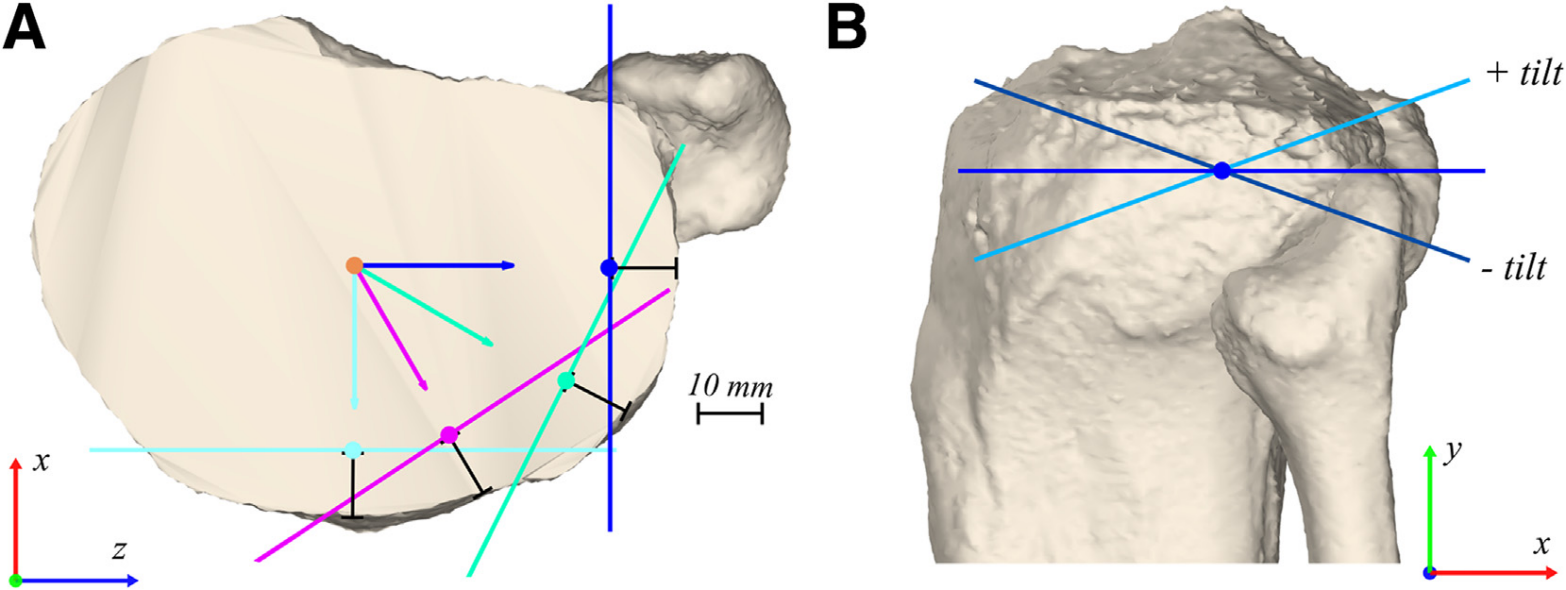
Stepwise change in the hinge axis (HA) direction through rotation and tilt in the axial plane. (A) Illustration of 4 of the 1° incremental steps of HA rotation in the axial plane. The HA was externally rotated between 0° (starting position: perpendicular to the tibial coronal plane; dark blue line) and 90° (parallel with the tibial coronal plane; light blue line), with the HA origin always at a distance of 10 mm to the tibial cortex. (B) Illustration of 3 of the 1° incremental steps of HA tilt around its origin relative to the axial plane. The HA was tilted between +20° (light blue line) and –20° (dark blue line). Positive and negative tilt were defined as the upslope (light blue) and downslope (dark blue) of the HA, respectively.

First, the HA was placed with its origin at a fixed position along the longitudinal (mechanical, y-) axis, at a fixed distance to the lateral tibial cortex of 10 mm, and with a direction perpendicular to the tibial coronal plane (and, therefore, parallel to the tibial sagittal and axial plane). Second, the HA direction was manipulated in the tibial axial plane by externally rotating it between 0° (perpendicular to the tibial coronal plane; Fig 3A, dark blue line) and 90° (perpendicular to the tibial sagittal plane; Fig 3A, light blue line) with 1° incremental steps (HA rotation; Fig 3; Table 1). This, effectively, leads to simulations of a medial, posteromedial, and posterior opening-wedge osteotomy, dependent on the HA orientation relative to the coronal and sagittal plane. For every external rotation of the HA, the HA origin was updated to remain at a distance of 10 mm perpendicular to the tibial cortex, which, therefore, guarantees a clinically relevant^36^ position (Fig 3A). This effectively caused the HA origin to move further anteriorly along the anterolateral cortex for every increase in external rotation. These steps were repeated with the same starting position of the HA origin and orientation but with additional tilt of the HA direction around its origin relative to the axial plane between –20° and +20° with 1° incremental steps (HA tilt; Fig 3; Table 1).^22^ Positive and negative tilt were defined as upslope (Fig 3B, light blue line) and downslope (Fig 3B, dark blue line), respectively. After each stepwise change in the HA direction, an opening-wedge high tibial osteotomy was simulated with the predefined 10° opening angle, and the coordinate system was updated according to the changed landmark positions. The tibial alignment parameters were then projected on their respective anatomical reference planes, and their values were calculated and visualised in a 3D plot using MATLAB r2021b.

**Table 1:** Overview of Parameters Defining the HA Orientation (Direction and Origin) and Their Possible Values

HA Parameters	Stepwise Change of HA Direction	Stepwise Change of HA Origin
Rotation in axial plane	0° to 90°; 1° steps	0° to 90°; 1° steps
Tilt in axial plane	–20° to 20°; 1° steps	–20° to 20°; 1° steps
Position along longitudinal axis	Tip fibula	Tip (0 mm) to circumference line fibula (14 mm); 2-mm steps
Distance to cortex	10 mm	5 to 10 mm; 1-mm steps

HA, hinge axis.

### Stepwise Change in the Position of the HA Along the Longitudinal Axis and the Distance of the HA Origin to the Tibial Cortex

**Appendix Fig 3:**
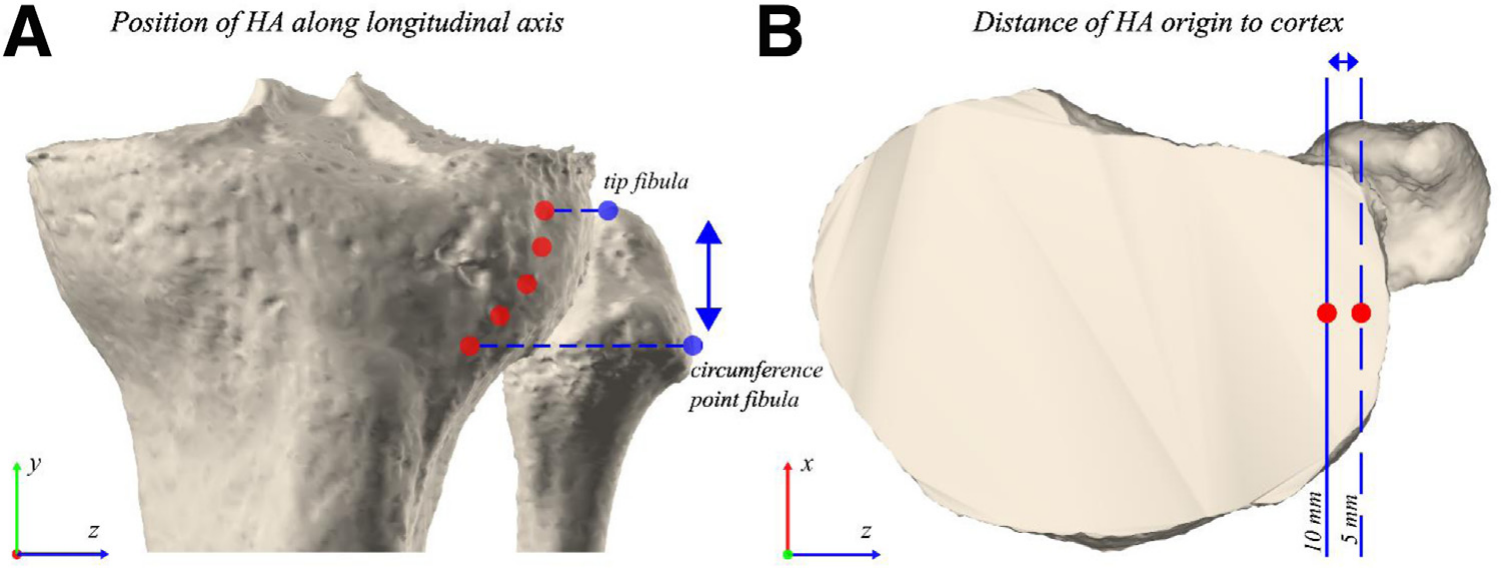
Stepwise change in position along the longitudinal axis and distance to the tibial cortex. (A) Anterior view of the proximal tibia and fibula with the position of the HA origin (red) shown moving along the longitudinal axis in 2-mm incremental steps from tip fibula (0 mm) to circumference point fibula (14 mm) while remaining at a set distance from the lateral tibial cortex. (B) Top view of the proximal tibia and fibula with the HA (blue) and origin (red) shown moving from a 10-mm (solid) to 5-mm (dashed) distance to the lateral tibial cortex in 1-mm incremental steps. (HA, hinge axis.)

In order to reflect real-life surgical circumstances, the position of the HA origin was manipulated along the longitudinal axis between the tip and the circumference point of the fibular head^36^ with incremental steps of 2 mm (which, in this tibia, was between 0 and 14 mm; Table 1; Appendix Fig 3). Furthermore, the distance to the tibial cortex of the HA origin was manipulated between 5 and 10 mm with incremental steps of 1 mm (Table 1; Appendix Fig 3). For all stepwise changes, the simulation and calculations described previously were repeated.

### Statistical Analysis

Since change in alignment parameter values were obtained mathematically through geometry over a range of parameters (i.e., HA rotation, HA tilt, HA position along longitudinal axis, and HA origin distance to the tibial cortex), no statistical analysis was performed.

## Results

Rather than presenting the actual alignment parameter values, data are presented as the difference between the alignment parameter value before and after the opening of the osteotomy to ensure greater clarity regarding the effect of every stepwise change in HA orientation in 3D space on alignment in all 3 anatomical reference planes.

### Effect of a Stepwise Change in HA Direction

**Fig 4:**
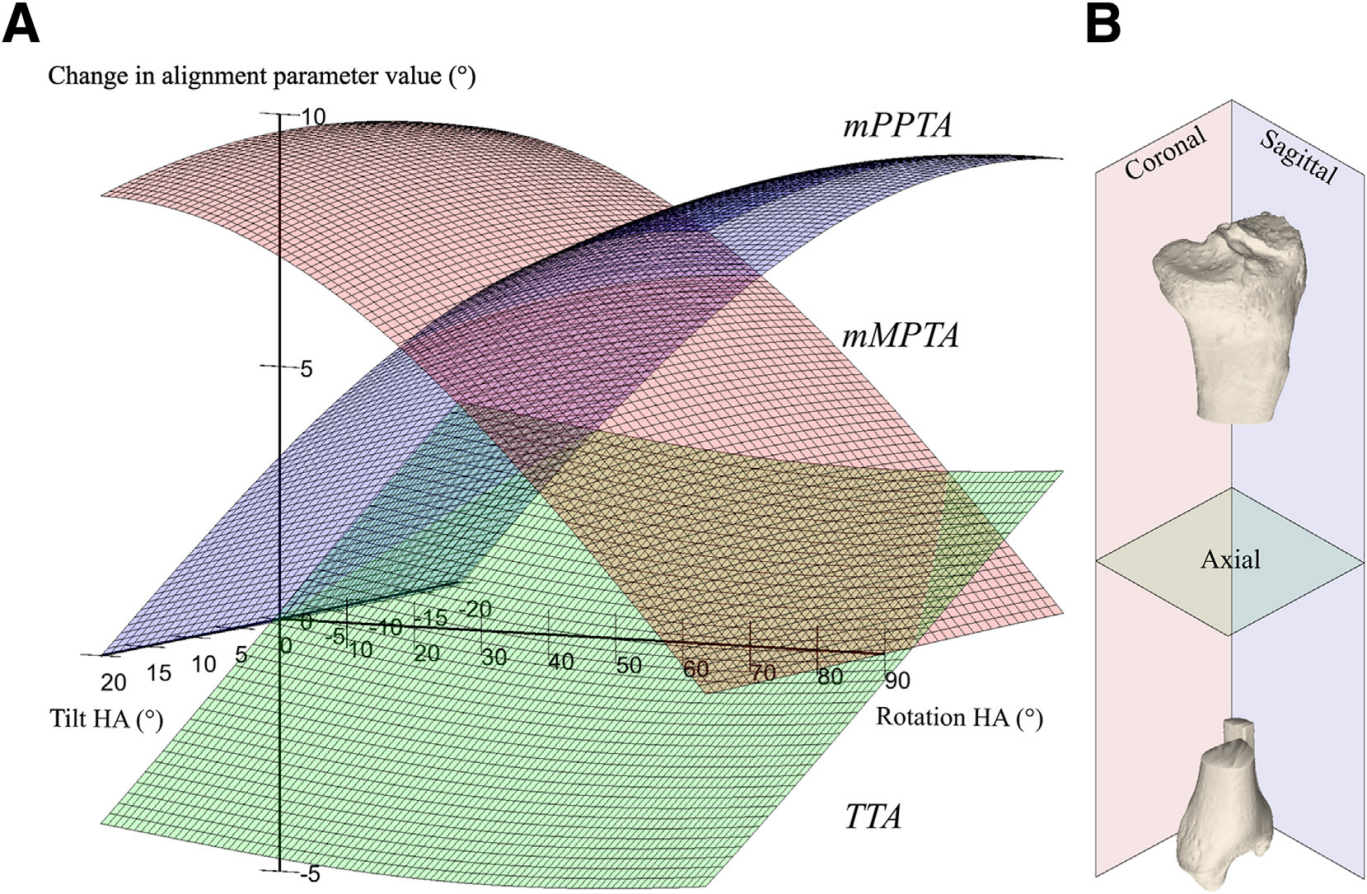
Three-dimensional visualization of the effects of hinge axis rotation and tilt on tibial alignment parameters. Effects are described by change in alignment parameter per anatomical reference plane after a simulated opening wedge osteotomy of 10°. An increase in mechanical medial proximal tibial angle (mMPTA; valgus), mechanical proximal posterior tibial angle (mPPTA; tibial upslope), and tibial torsional angle (TTA; external rotation) were defined as positive. Positive hinge axis (HA) tilt was defined as upslope, and HA rotations of 0° and 90° were defined as perpendicular to the tibial coronal and sagittal planes, respectively. (A) An HA rotation between 0° and 90° with 0° of HA tilt has a major effect on both tibial coronal (mMPTA, red) and sagittal plane (mPPTA, blue) alignment, and this effect is inverse between these planes. HA rotation has only a minor effect on axial plane (TTA, green) alignment. By contrast, additional HA tilt between –20° and 20° has major effects on tibial axial alignment, while it has only minor effects on both coronal and sagittal plane alignment. (B) The tibia/fibula anatomical reference planes correspond to the color of the alignment parameter that is measured in that plane.

**Fig 5:**
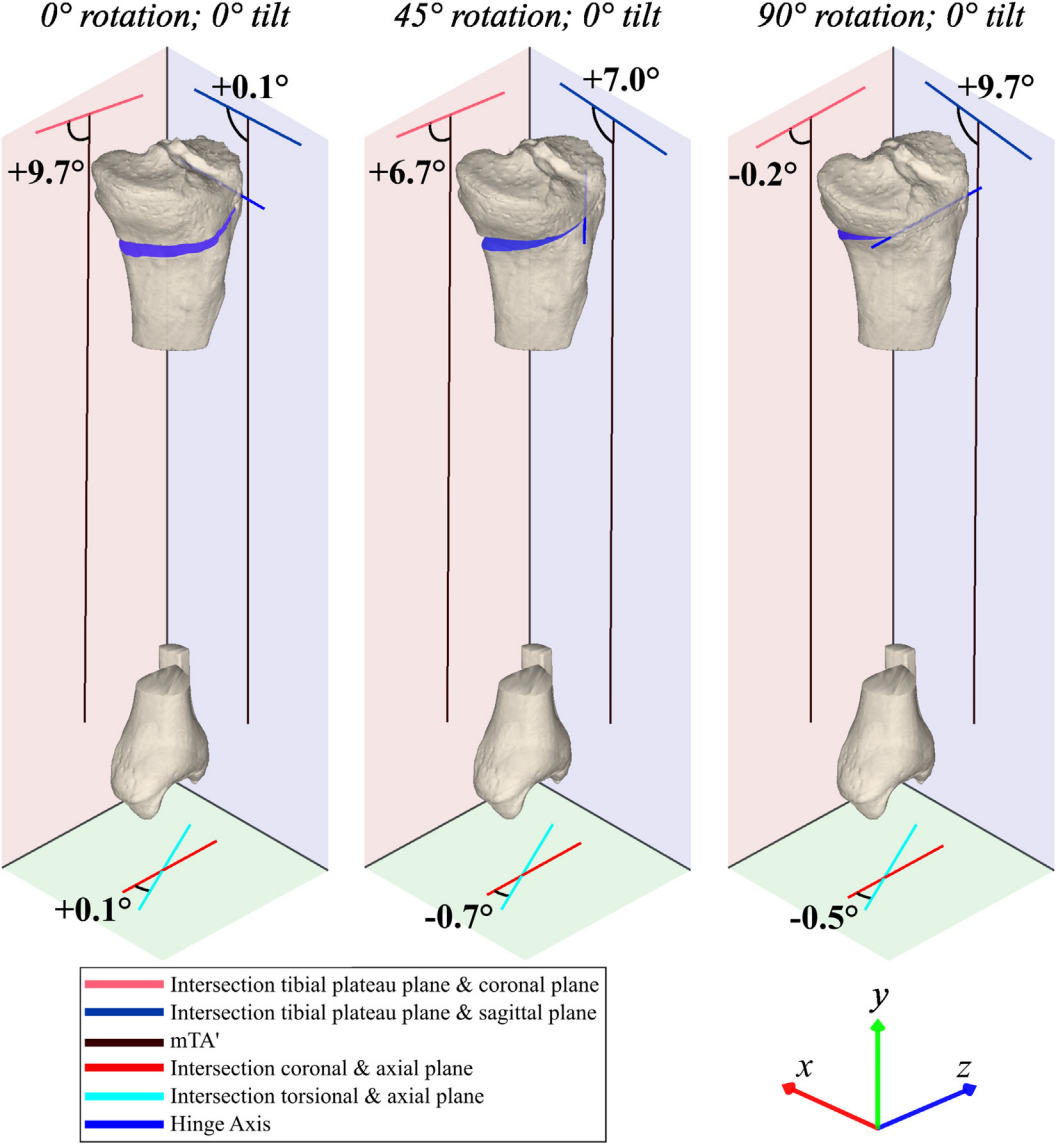
An illustrative selection of the effect of hinge axis rotation (in the axial plane). Simulated opening-wedge osteotomies of 10° on the same proximal tibia at 0° hinge axis (HA) rotation (left), 45° HA rotation (middle), and 90° HA rotation (right). The HA tilt was always kept at 0°. HA rotation has major effects on both tibial coronal (red) and sagittal (blue) plane alignment, and this effect is inverse between these planes, while it has only minor effects on axial plane alignment (green). The HA and osteotomy planes are shown in dark blue. (mTA’, projected mechanical tibial axis.)

**Fig 6:**
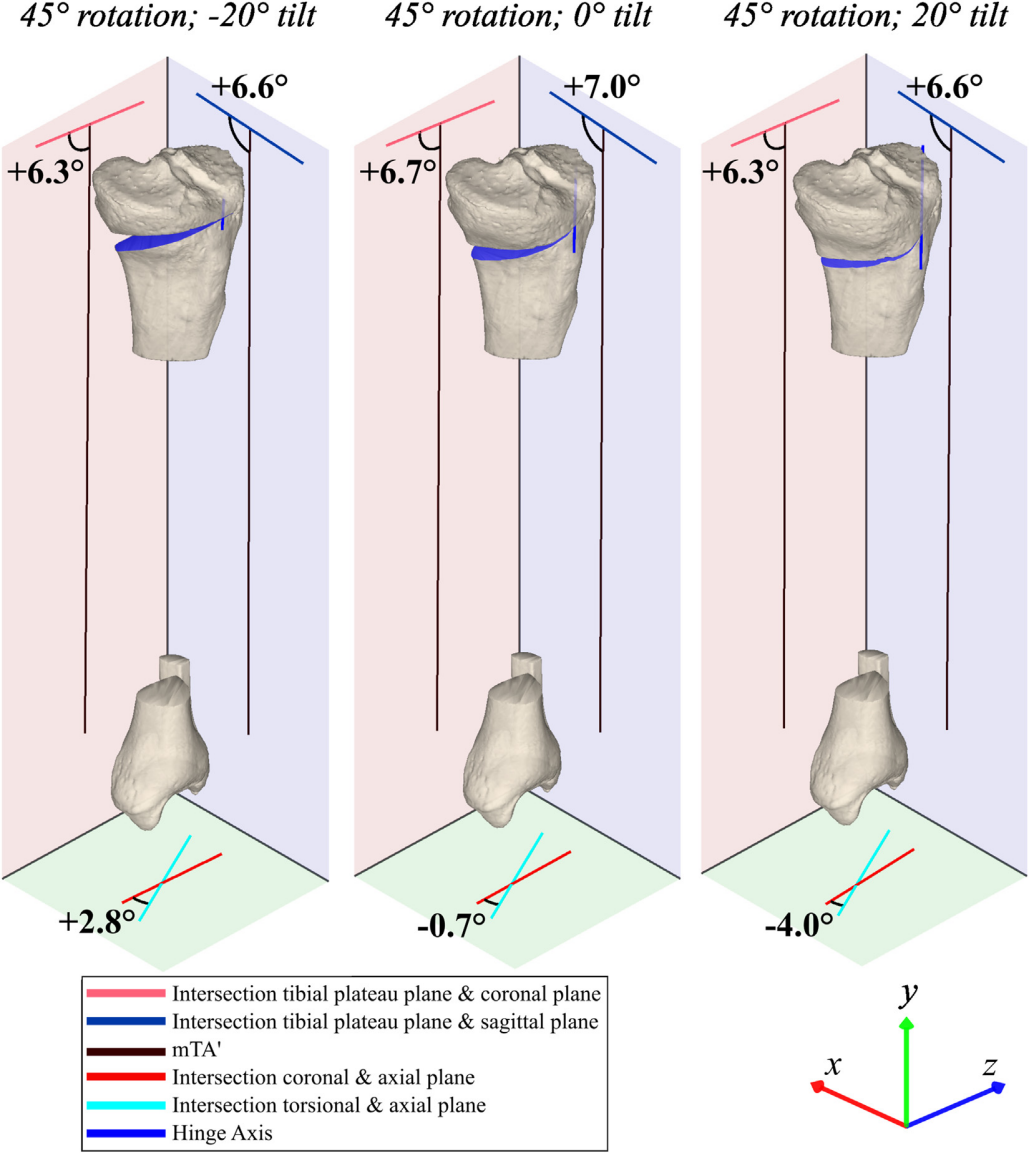
An illustrative selection of the effect of hinge axis (HA) tilt (relative to the axial plane). Simulated opening wedge osteotomies of 10° on the same proximal tibia at –20° HA tilt (left), 0° HA tilt (middle), and 20° HA tilt (right). The HA rotation was always kept at 45°. HA tilt has major effects on tibial axial plane (green) alignment, while it has only minor effects on coronal (red) and sagittal (blue) plane alignment. The HA and osteotomy planes are shown in dark blue. (mTA’, projected mechanical tibial axis.)

The effects of all possible combinations of HA rotation and HA tilt after opening the osteotomy by 10° are shown in Figure 4. An illustrative selection of the separate effects of HA rotation and HA tilt on tibial coronal, sagittal, and axial plane alignment after opening the osteotomy by 10° is shown in Figures 5 and 6, which depict 3 selected HA rotations and tilts, respectively. A HA rotation between 0° (perpendicular to the coronal plane) and 90° (perpendicular to the sagittal plane) with a 0° HA tilt primarily affected change in alignment within the coronal (mMPTA) and sagittal planes (mPPTA), with an inverse effect between these planes. With the HA rotated 0°, the change in coronal plane alignment (mMPTA) was 9.7°, whereas the change in sagittal plane alignment (mPPTA) was 0.1°. Inversely, with the HA rotated 90°, the change in sagittal plane alignment (mPPTA) was 9.7°, whereas the change in coronal plane alignment (mMPTA) was 0.2°. The effect of HA rotation on the change in axial plane alignment (tibial torsional angle) was maximally 0.9°. In contrast, HA tilt between –20° and +20° primarily affected change in axial plane alignment (tibial torsional angle; max. Δ6.9°). The effect of HA tilt on the change in coronal alignment (mMPTA) and sagittal alignment (mPPTA) was maximally 0.6°.

### Effect of Stepwise Change on the Position of the HA Origin Along the Longitudinal Axis and the Distance From the HA Origin to the Tibial Cortex

The change in position of the HA origin along the longitudinal axis (0-14 mm) and the change in distance from the HA origin to the cortex between 10 mm and 5 mm only had a minor effect on alignment in any of the anatomical reference planes (max. Δ0.03°-0.3°, and max. Δ0.008°, respectively).

## Discussion

The most important findings of the present study are that, when performing an opening-wedge high tibial osteotomy, HA rotation between orientations perpendicular to the coronal and sagittal planes primarily affects the change in both tibial coronal and sagittal plane alignment, and this effect is inverse between these planes. In contrast, HA tilt relative to the tibial axial plane primarily affects the change in tibial axial alignment. These findings support our hypotheses.

The results show a clear (and expected) coupling between the orientation of the HA in 3D space and its effect on alignment parameter values in all 3 anatomical reference planes. Although similar effects of HA orientation on alignment in 2 of the 3 anatomical reference planes as the ones reported in the present study have been reported before,^18^, ^19^, ^20^^,^^22^^,^^23^^,^^37^ Jörgens et al.^31^ investigated the effects of HA rotation and HA tilt on alignment angles in all 3 anatomical reference planes. However, Jörgens et al.^31^ investigated this effect to explain why control of HA orientation can minimize unintended changes in alignment in nontargeted anatomical reference planes when performing uniplanar malalignment correction osteotomies, with a small range of angled HA orientations. By contrast, the present study investigated the effect of HA orientation to also explain how to facilitate intentional corrections in all anatomical reference planes, with a larger range of angles. When transitioning from 2D to 3D imaging and surgical technologies, this insight can be used to enforce simultaneous changes in alignment in all anatomical reference planes with one osteotomy.

Interestingly, Jörgens et al.^31^ used a coordinate system based on an axial plane that was defined by the tibial plateau’s articular surface. However, the tibial plateau is tilted (and not orthogonal) relative to the mTA. Since the indication for an osteotomy around the knee is to change mechanical (mal)alignment,^33^ the simulations in the present study were performed in a coordinate system based on the mechanical tibial axis, and the results show that the coordinate system with tilt relative to the mTA as used by Jörgens et al.^31^ would have introduced an unintended change in axial plane alignment. Therefore, in order to use HA orientation for control of the simultaneous change of mechanical alignment in all anatomical reference planes, planning and execution of the osteotomy relative to a coordinate system based on mechanical axes, as proposed by Paley,^34^ is advised.

The results of the present study may have several clinical implications for the commonly used 2D imaging–based framework for osteotomies around the knee. First, it is plausible that the reliance on 2D fluoroscopy to align the osteotomy plane in coronal alignment-correcting osteotomies introduces unintended HA rotation and tilt, which could explain the inability to reliably obtain the targeted correction and the occurrence of unintended corrections,^12^^,^^15^^,^^21^ For example, with 2D fluoroscopy, it is not possible to intraoperatively align the X-ray beams perpendicular or parallel to the mTA and, therefore, to any of the anatomical reference planes of a clinically relevant coordinate system based on a mechanical axis.^35^ In addition, alignment analysis and correction planning are performed on a 2D long-leg radiograph. In a later stage, malalignment correction is executed using 2D fluoroscopy of the proximal tibia. Possible differences in position of the tibia relative to the X-ray beam and detector between the preoperative long-leg radiograph and the 2D fluoroscopy during surgery lead to rotation of the depicted “coronal plane”; therefore, the introduction of a HA rotation is likely to occur. Furthermore, 2D fluoroscopy is used to align the osteotomy cutting plane parallel to the tibial plateau’s anteroposterior articular surface. However, this articular surface is not perpendicular to the mTA.^38^ Instead, it has a sagittally oriented downslope, thereby introducing a (negative) HA tilt of several degrees relative to the mTA (and, therefore, the coordinate system’s axial plane).

Second, in high tibial osteotomy procedures aimed at changing sagittal plane alignment, unintended HA rotation also might be introduced. In these procedures, sagittal plane analysis, correction planning, and execution are all performed using a 2D lateral knee radiograph. On a 2D lateral knee radiograph, the sagittal plane is defined by the overlap of the medial and lateral posterior tibial condyles (defining the direction of the posterior tibial condylar axis) and the medial and lateral tibial plateaus. The mechanical sagittal plane, however, is defined as perpendicular to the line that connects the most medial and lateral points of the medial and lateral tibial plateaus, respectively (in the present study: proximal tibial joint orientation medial–lateral) and parallel to the mTA.^35^ When projected on the coordinate system’s axial plane, the posterior tibial condylar axis is not parallel to the proximal tibial joint orientation medial–lateral. Therefore, on 2D radiographs, the depicted “sagittal plane” is different from that of a clinically relevant coordinate system based on the mTA.

Thus, these 2 types of high tibial osteotomy procedures based on a 2D-framework inherently introduce unintended HA rotation and HA tilt and, therefore, uncontrolled corrections in coronal, sagittal, and axial plane alignment. To place this in a clinical perspective, a rotation of the “anteroposterior” fluoroscopy image relative to the real coronal plane^21^ between 10° internal and external rotation in a 10° medial opening wedge high tibial osteotomy would lead to unintended sagittal corrections of +1.65° and –1.65°, respectively (and therefore to an unintended difference of 3.3°), and only 9.3° (instead of 10°) of coronal plane correction. Moreover, alignment of the osteotomy plane parallel to the tibial plateau on an anteroposterior fluoroscopy image, as is common practice (a proxy for HA tilt relative to the mTA of –10°),^39^ would lead to an unintended difference in axial correction of 1.5°. While it is currently unknown whether such values of imprecision affect clinical outcomes significantly, the cumulative imprecision in all 3 dimensions might lead to the reported unintended corrections in nontargeted planes in the execution of uniplanar osteotomies.^12^^,^^40^ Nonetheless, these observations might render 2D fluoroscopy insufficient if control of HA orientation in 3D space is pursued to prevent unintended corrections in nontargeted anatomical reference planes, or to simultaneously change alignment in all 3 anatomical reference planes of a clinically relevant coordinate system.

Last, it has been reported that the HA orientation attained during high tibial osteotomy procedures with a 2D framework can be negatively influenced by the triangular shape of the proximal tibia, the posteromedial soft tissues (medial collateral ligament, medial hamstrings tendons), and the proximal tibiofibular joint (which serves as a natural hinge) posterolaterally,^41^^,^^42^ causing a posterolaterally or posteromedially oriented HA (i.e., unintended HA rotation in the axial plane). Using the bone’s plasticity to change the osteotomy’s opening-gap ratio^41^^,^^43^ after malorientation of the HA or performing a posteromedial opening wedge instead of a medial opening wedge high tibial osteotomy to “blindly” influence HA orientation^44^ have been proposed as solutions to this issue. However, enforcing 3D HA orientation to explicitly achieve simultaneous correction in all three anatomical reference planes must involve the use of a 3D framework incorporating both 3D alignment analysis and planning and a one-on-one translation of the planned HA orientation to the executional phase of the osteotomy. This can be achieved using imaging techniques such as CT and MRI to render 3D bone models and 3D surgical techniques such as patient-specific guides, computer navigation, and/or robot-assisted surgery to execute the planned alignment correction.

The clinical relevance of this study is that it shows that integrating 3D HA orientation in malalignment correction planning and execution offers the potential to minimize unintended corrections in nontargeted planes in uniplanar correction osteotomies, and to facilitate intentional multiplanar correction with a single osteotomy. To indicate the clinical application, we describe how the results of the present study are applied in the practice of the senior author orthopaedic surgeon to perform multiplanar correction osteotomies. Using the presented results, the intended corrections in all anatomical reference planes can be defined. Subsequently, with an in-house developed MATLAB script, the corresponding 3D orientation of the HA and the opening or closing angle of the osteotomy are calculated—usually with a HA rotation between 55° and 85° depending on the amount of varus and slope correction in the particular patient—within a predefined surgically viable operating window, and with a predefined distance from the HA origin to the cortex. The obliquity of the osteotomy plane can be adapted manually to situate it in a surgically feasible location. While in this virtual study, the position of the HA origin along the longitudinal axis at the level of the proximal tibiofibular joint had a negligible effect on alignment in all three anatomical reference planes, in reality, it might be necessary to avoid this natural posterolateral hinge by planning the osteotomy plane at or just above the tip of the fibular head, just outside the proximal tibiofibular joint capsule. Then, a patient-specific saw guide is designed with which the HA orientation and osteotomy plane can be enforced during the osteotomy procedure. In order to negate the effect of soft tissue effect on the opening angle of the osteotomy, the eventual positions of the screws of the osteotomy-stabilizing fixation plate are incorporated in the guide design.

### Limitations

This study has several limitations. First, the experiment was performed on one tibial 3D bone model only. Second, the virtual simulation in the present study assumes that structures both lateral and medial to the HA rotate equally around the HA. In vivo, however, plastic deformation of the bone or soft-tissue tension could influence the change in alignment, even when the planned HA orientation is correctly achieved. Third, only an opening-wedge high tibial osteotomy, and no other approaches, such as a closing-wedge high tibial osteotomy or a distal femoral osteotomy, were simulated. However, it is probable that the principles that dictate an osteotomy as presented in this study apply to other osteotomy types and that using a closing instead of an opening wedge technique would lead to a more or less equal, but inverse, change in alignment angles.

## Conclusions

HA rotation in the tibial axial plane primarily affects sagittal and coronal plane alignment, and HA tilt relative to the tibial axial plane primarily affects axial plane alignment.

## Disclosure

The authors (Q.W.T.V., R.M.t.H., G.J.M.T., F.d.G., R.F., R.A.G.H.) report no conflicts of interest in the authorship and publication of this article. Full ICMJE author disclosure forms are available for this article online, as supplementary material.

## Figures and Tables

**Appendix Fig 1:**
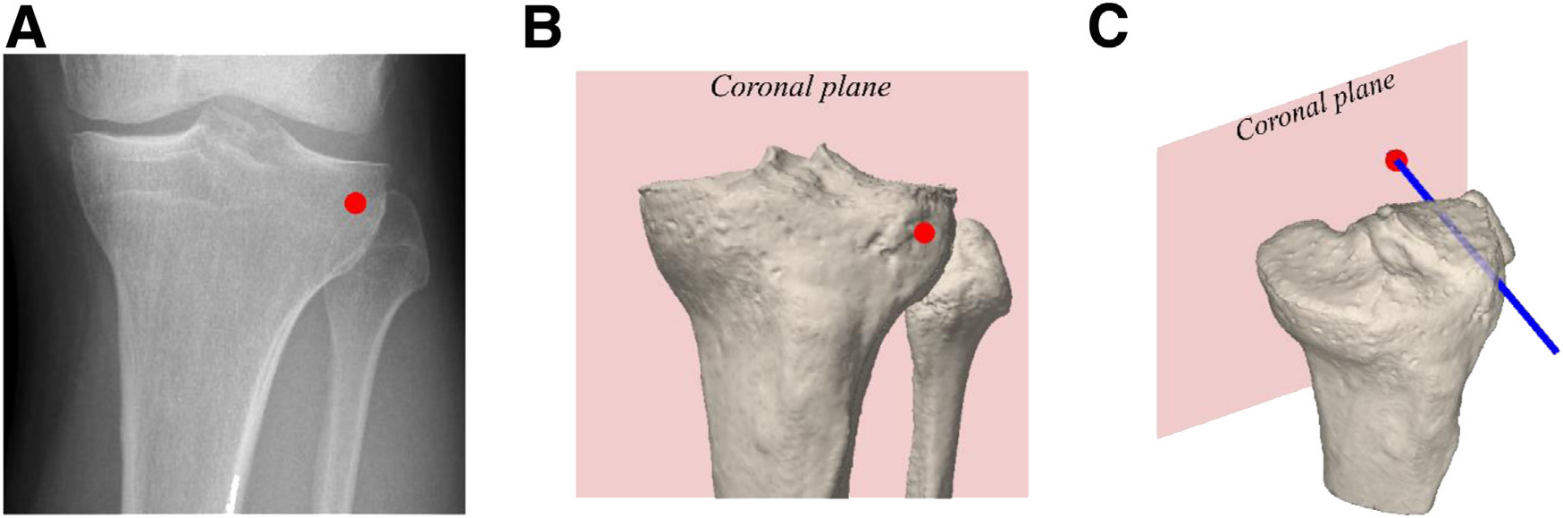
The relationship between the hinge point and the hinge axis. In conventional osteotomy correction planning and execution, a hinge point over which the osteotomy rotates is positioned on a 2-dimensional radiograph (A), which is a projection of a 3-dimensional bone on one 2-dimensional plane (B). In a 3-dimensional perspective, however, it is clear that this hinge point is actually a hinge axis positioned perpendicular to a radiologically depicted 2-dimensional anatomical reference plane (C).

## Supplementary Data

ICMJE author disclosure forms
